# Evaluation of computer-tailored health education (‘E-health4Uth’) combined with personal counselling (‘E-health4Uth + counselling’) on adolescents’ behaviours and mental health status: design of a three-armed cluster randomised controlled trial

**DOI:** 10.1186/1471-2458-12-1083

**Published:** 2012-12-17

**Authors:** Rienke Bannink, Evelien Joosten - van Zwanenburg, Petra van de Looij - Jansen, Els van As, Hein Raat

**Affiliations:** 1 Department of Public Health, Erasmus MC University Medical Center Rotterdam, P.O. Box 2040, 3000 CA, Rotterdam, the Netherlands; 2 Municipal Public Health Service South-Holland South, Karel Lotsyweg 40, 3318 AL, Dordrecht, the Netherlands; 3 Municipal Public Health Service Rotterdam area, Schiedamsedijk 95, 3011 EN, Rotterdam, the Netherlands; 4 Department of Youth Health Care, Rivas zorggroep, P.O. Box 90, 4200 AB, Gorichem, the Netherlands

**Keywords:** Adolescents, Youth, Health behaviour (alcohol, drugs, smoking, safe sex), Psychosocial problems, Mental health, Well-being, E-health4Uth, Tailored health education, Counselling, Tailored counselling

## Abstract

**Background:**

About 15% of adolescents in the Netherlands have mental health problems and many also have health risk behaviours such as excessive alcohol consumption, cigarette smoking, use of drugs, and having unsafe sex. Mental health problems and health risk behaviours may have adverse effects on the short and longer term. Therefore, in the Netherlands there is a considerable support for an additional public health examination at age 15–16 years. The study evaluates the effect of two options for such an additional examination. Adolescents in the ‘E-health4Uth’ group receive internet-based tailored health messages on their health behaviour and well-being. Adolescents in the ‘E-health4Uth + counselling’ group receive the computer-tailored messages combined with personal counselling for adolescents at risk of mental health problems.

**Methods and design:**

A three-arm cluster randomised controlled trial will be conducted in the Netherlands among fourth-grade secondary school students. School classes are the unit of randomisation. Both intervention groups complete the computer-tailored program during one class session; the program focuses on nine topics related on health behaviour and well-being. For each topic a score is computed that can be compared with the Dutch health norms for adolescents. Based on the score, a message is presented that reflects the person’s current behaviour or well-being, the Dutch health norm, and offers advise to change unhealthy behaviour or to talk to a person they trust. Adolescents in the ‘E-health4Uth + counselling’ group are also invited for an appointment to see the nurse when they are at risk of mental health problems. The control group receives ‘care as usual’.

The primary outcome measures are health behaviour (alcohol, drugs, smoking, safe sex) and mental health status. The secondary outcome measure is health-related quality of life. Data will be collected with a questionnaire at baseline and at 4-months follow-up. A process evaluation will also be conducted.

**Discussion:**

It is hypothesized that at follow-up adolescents in the ‘E-health4Uth’ group and adolescents in the ‘E-health4Uth + counselling’ group will show fewer mental health problems and less risky behaviour compared to the control group.

**Trial registration:**

Current Controlled Trials NTR3596

## Background

### Problems among adolescents

About 15% of adolescents in the Netherlands have mental health problems [1]. During adolescence, mental health problems among girls tend to increase, and young people from lower educational levels or from families with a lower socioeconomic status report more difficulties than young people from better-off families [1,2]. Young persons with mental health problems are at risk of adverse mental health outcomes later on [3-5]. In general, high levels of behavioural and emotional problems at a young age are related to the DSM-IV diagnoses in adulthood [6].

Health risk behaviours such as excessive alcohol consumption, cigarette smoking, use of drugs, and having unsafe sex are also prevalent among adolescents [1]. The Health Behaviour in School-Aged Children: WHO Collaborative Cross-National Study (HBSC) shows that of the 16-year-old adolescents in the Netherlands about 71% drank alcohol during the 30 days preceding the survey, 19% smoked daily, 12% used cannabis during the 30 days preceding the survey, and 20% of the sexually active adolescents did not use a condom at last sexual intercourse [1]. These health risk behaviours may have adverse effects on the short and longer term [7]. For example, through unsafe sex adolescents can get sexually transmitted diseases or become pregnant [1]. Beginning at an early age with alcohol use may have adverse psychological consequences [8] and smoking may negatively affect school performance [9]. Moreover, alcohol and drugs use are often associated with aggressive and delinquent behaviour [10-12].

### Early detection and prevention of problems in adolescence

The Netherlands has a well-organised system for maintenance of the health of children, i.e. the Youth Health Care system [13]. All children and adolescents are invited for ‘preventive periodic health examinations’ at set ages until the age of 13 years. These examinations focus on growth, development, health functioning, and behaviour of infants, children, and adolescents. At age 13 years, the physician or nurse interviews the adolescents and provides health counselling. If physical of psychosocial problems are encountered, the physician or nurse may invite the adolescent (and parents) for a follow-up visit or may refer them to a general practitioner. Participation is voluntary and the care is offered free of charge by the government.

Given the rapid maturation of adolescents, and the associated mental health problems and health risk behaviours, there is a considerable support in the Netherlands for an additional examination at age 15–16 years. Several options for this examination have been proposed by the National Institute for Public Health and Environment [14]. One option is that adolescents complete an online questionnaire about their health behaviour and well-being, and then receive tailored messages about their health behaviour and well-being (‘E-health4Uth’). Computer-tailored health education is a good opportunity to provide health messages regarding individual health behaviour and behavioural determinants, because tailored messages eliminate (as far as possible) information that is not personally relevant [15-18]. Computer-tailored messages are more likely to be effective in changing behaviour compared to non-tailored messages [16]. A second option, advised by the National Institute for Public Health and Environment, is to combine the computer tailoring with personal counselling for adolescents at risk of mental health problems (‘E-health4Uth + counselling’) [14]. The online questionnaire used for generating the tailored messages can also be used for early detection of adolescents at risk of mental health problems. Early detection of mental health problems, and if necessary tailored counselling or referral for treatment, may improve the prognosis of adolescents with mental health problems [19]. Therefore, it may be beneficial to combine computer tailoring with personal counselling for adolescents at risk of mental health problems.

For both these options (‘E-health4Uth’ and ‘E-health4Uth + counselling’), it is possible to use E-health modules with internet-based tailored messages, which were developed for adolescents (aged 12–18 years) and applied in an earlier study [20]. In a process-evaluation it was found that the individually-tailored messages were appreciated by adolescents. However, the effects of the internet-based messages on adolescents’ behaviour and well-being has not yet been studied [21].

### Objectives

The aim of this study is to evaluate the effect of two interventions on mental health and health behaviour (alcohol and drug use, smoking, safe sex). Adolescents in the intervention group ‘E-health4Uth’ receive the computer-tailored health education. Adolescents in the intervention group ‘E-health4Uth + counselling’ receive computer-tailored health education combined with counselling for adolescents at risk of mental health problems. Additionally, a process evaluation will be conducted to provide insight in the feasibility of the two interventions. This article describes the design of the study.

### Study hypothesis

The hypotheses of the study are twofold. First, adolescents in the ‘E-health4Uth’ group will show fewer mental health problems and less risky behaviour (alcohol and drug use, smoking, safe sex) at follow-up compared to the control group (‘care as usual’). Second, adolescents in the ‘E-health4Uth + counselling’ group will show fewer mental health problems and less risky behaviour (alcohol and drug use, smoking, safe sex) at follow-up compared to the control group (‘care as usual’).

## Methods and design

### Study design

The study design is a three-armed cluster randomised controlled trial (RCT), with a baseline measure point before the interventions and a follow-up measure four months after start of the interventions. This study is conducted in the Netherlands among fourth-grade secondary school students (aged 15–16 years). School classes are the unit of randomisation, because randomisation at the individual level (i.e. the level of the adolescents) may lead to contamination of the control group [22]. Block randomisation is used as this allows to start the study whilst still including schools and classes. The randomisation code was developed using a computer random number generator in SPSS to select random permuted blocks (specified allocation ratio 1:1:1). School classes are stratified according to educational level, with pre-vocational education in one stratum and higher than pre-vocational education in the other stratum.

The effects of ‘E-health4Uth’ and ‘E-health4Uth + counselling’ will be evaluated at 4-months follow-up by comparing the outcomes on mental health status and health behaviour between the adolescents in the two intervention groups and in the control group.

Data collection started in the autumn of 2012 and will continue until June 2013. The Medical Ethical Committee of Erasmus MC declares that the Medical Research Involving Human Subjects Act (also known by its Dutch abbreviation WMO) does not apply to this research proposal. The Medical Ethical Committee has no objection against the execution of this research proposal (MEC-2012 – 337).

### Procedure

A few weeks prior to the start of the study, all adolescents and parents receive information about the study. Information letters providing details about the study for the students and the parents are handed out at school. If parents do not want their child to participate, they can object to participation of their child. Adolescents are asked for their permission to participate before they complete the baseline questionnaire.

All adolescents receive a singular, personal code to log in at one of the three websites of the study. The link of the websites depends on the group (‘E-health4Uth’, ‘E-health4Uth + counselling’, or the control group) to which the adolescent is assigned. At the websites the adolescents can complete the questionnaires. A trained research assistant explains the procedure for the completion of the questionnaire at the beginning of the lesson and is available to answer questions. During one class session (+/− 45 min) adolescents complete the online questionnaire. In the two intervention groups the adolescents also receive the tailored messages during this class session. Prior to the start of the study, specialised Youth Health Care nurses were informed about the study and trained in interviewing adolescents in this age group.

**Figure 1:**
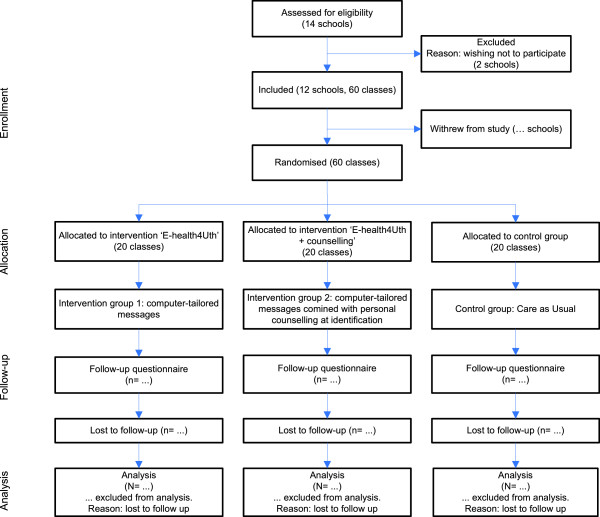
Flow chart of the clusters and participants through the trial.

The participant flow is shown in Figure 1.

### Participants

#### Youth Health Care organisations and schools

This study is carried out in the Dutch cities of Dordrecht and Zwijndrecht. Recruitment of schools is organised in collaboration with two Youth Health Care organisations. The directors of 14 secondary schools in Dordrecht and Zwijndrecht have been informed about the study by letter, invited to participate, and have been contacted by the researchers. From the 14 schools, 12 agreed to participate in the study with a total of 60 classes of fourth-grade students.

#### Adolescents

Per school, all the students in the fourth grade are invited to participate. A trained researcher assistant briefly explains the purpose and procedure of the study in the classroom.

### Intervention ‘E-health4Uth’

Adolescents complete the computer-tailoring program during one class session (+/− 45 min). This program focuses on nine topics related to health risk behaviour and well-being: alcohol consumption, drugs use, smoking, sexual behaviour, bullying, mental health status, suicidal thoughts, suicidal attempts and unpleasant sexual experience (Table 1). We use internet-based tailored messages which were developed for adolescents (aged 12–18 years) and applied in an earlier study [21]. The questionnaire used for the tailored messages is constructed on the basis of several existing instruments which are used by Municipal Public Health Services and health institutes (Table 1) [23]. Consensus on the use of these instruments was established by the National Institute for Public Health and Environment (RIVM), the Dutch association for residential and home care organisations and infant and child health clinics (Actiz), and the Association of Muncipal Public Health Services in the Netherlands (GGD Nederland).

**Table 1:** Topics of the E-health modules

**Behaviour and well-being**	**Items**
Alcohol consumption	How often and how much the adolescent drinks (9 items)
Drugs use	How often the adolescents has used different types of drugs (17 items)
Smoking	How often the adolescent smokes (2 items)
Sexual behaviour	How often the adolescent uses condoms during sexual intercourse (2 items)
Bullying	How often the adolescent is bullied at school, somewhere else or on the internet (3 items)
Mental health status	Strength and Difficulties Questionnaire (SDQ) (25 items) with a total score range 0 - 40
Suicidal thoughts	If the adolescent has had suicidal thoughts last year (1 item)
Suicidal attempts	If the adolescent made a suicidal attempt last year (1 item)
Unpleasant sexual experience	If the adolescent has ever had an unpleasant sexual experience (1 item)

**Figure 2:**
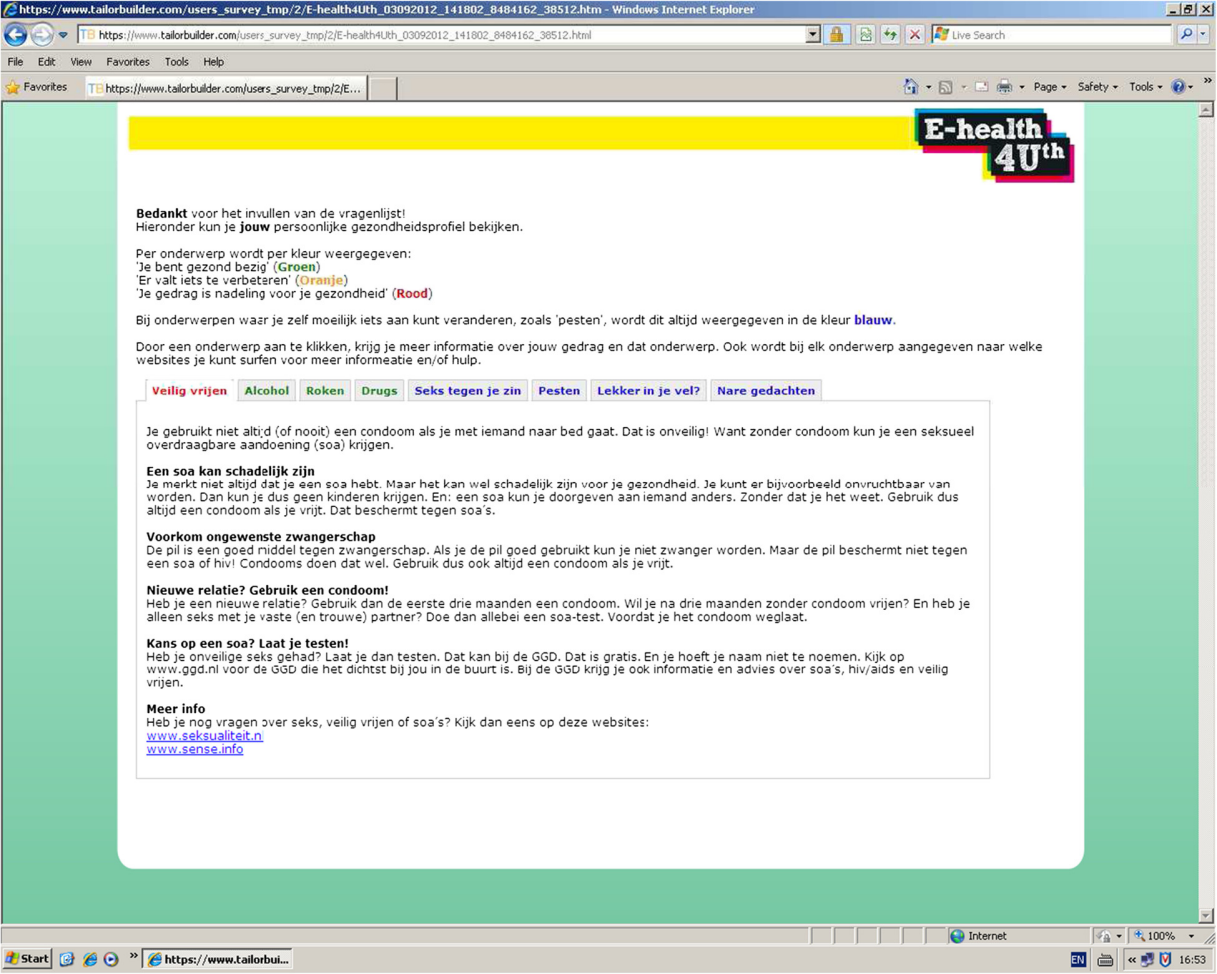
Screenshot of the ‘E-Health4Uth’ intervention.

For each topic, a score is computed that can be compared with the Dutch health norms for adolescents [21]. Based on the score, a message is presented that reflects the person’s current behaviour or well-being, the Dutch health norm, and offers advise to change unhealthy behaviour or to talk to a person they trust. By providing links to relevant websites, adolescents are encouraged to search for more information on the topics. The tailored messages are presented on the computer screen immediately after the questionnaire has been completed (Figure 2). The messages are displayed in red, orange or green, indicating unhealthy behaviour, behaviour just below the norm, or behaviour according to the Dutch health norm, respectively. The topics on well-being are always displayed in blue. At the end of the program, adolescents are invited to follow the Facebook page of ‘E-health4Uth’ to find additional information about all the topics. Also, adolescents can check a box for a self-referral with the nurse, or can send an e-mail to the nurse. After one month the adolescents receive a reminder of the tailored messages.

### Intervention ‘E-health4Uth + counselling’

Adolescents in this group also receive the tailored messages, are invited to follow the Facebook page (see Intervention ‘E-health4Uth’), and can check a box for a self-referral with the nurse or send an e-mail to the nurse. In this group, adolescents at risk of mental health problems are also invited for an appointment to see the nurse (Table 2). The criteria for an appointment are: those who report mental health problems as measured with the Strengths and Difficulties Questionnaire (SDQ) (cut-off point > 90^th^ percentile), those who report emotional problems as measured with the SDQ subcale ‘emotional problems’ (cut-off point > 90^th^ percentile), those who report having suicidal thoughts ‘occasionally’ or more often, and those who report a suicide attempt last year. The cut-off points of > 90^th^ percentile are based on a cross-national survey among 15–16 year old adolescents. This study was carried out by the Health Behaviour in School-Aged Children: WHO Collaborative Cross-National Study (HBSC) [1].

**Table 2:** Cutt-off points used to select adolescents at risk for mental health problems (in intervention group ‘E-health4Uth + counselling’)

**Well-being**	**Cutt-off point applied**
Mental health status (25 items)	Score on the Strength and Difficulties Questionnaire (SDQ) ≥ 17, or a score of ≥ 6 on the subscale emotionality of the SDQ [1]
Suicidal thoughts (1 item)	Having suicidal thoughts ‘occasionally’ or more often
Suicidal attempts (1 item)	Made an attempt at suicide last year

The appointment with the nurse takes place at the adolescent’s school. We expect that about 20% of the adolescents will meet the inclusion criteria for an appointment with the nurse [24]. The nurses receive the results of the assessment for each referred adolescent prior to the consultation. During the consultation the nurses focus on specific risk areas, and refer adolescents to other professionals when considered necessary.

### Control group

The control group receives care as usual, i.e. adolescents can check a box for a self-referral with the nurse or can send an e-mail to the nurse.

### Measurements

#### Primary outcome measures

The primary outcomes of the study are adolescents’ health behaviours (alcohol, drug, smoking, safe sex) and mental health status. The electronic, self-administered questionnaire, which is used for the tailored messages in the two intervention groups, serves also as the baseline questionnaire. The questionnaires used for the health behaviours are constructed on the basis of several existing instruments used by Municipal Public Health Services and health institutes [23] (Table 1). Mental health status is measured by the Strengths and Difficulties Questionnaire (SDQ) [25-29] and the Youth Self Report (YSR) [30]. The SDQ consists of 25 items describing positive and negative attributes of adolescents that can be allocated to 5 subscales of 5 items each: the emotional problems subscale, the conduct problems subscale, the hyperactivity-inattention subscale, the peer problems subscale, and the prosocial behaviour subscale. Each item is scored on a 3-point scale with 0=not true, 1=somewhat true, and 2=certainly true. A total difficulties score is calculated by summing the scores on the emotional problems, conduct problems, hyperactivity-inattention, and peer problems subscales (range 0–40) [26]. The YSR comprise 119 items addressing emotional and behavioural problems of adolescents. Respondents have to indicate on 3-point scales the extent to which each item applies: 0=not, 1=sometimes, or 2=often. The YSR assess two broad domains of psychopathology: one is the externalizing which reflects behavioural problems and the other is internalizing which refers to emotional problems. In addition, problems can be grouped into eight narrow-band scales: anxious-depressed, withdrawn-depressed, somatic complaints, social problems, thought problems, attention problems rule-breaking behaviour, and aggressive behaviour [30].

The health behaviours and SDQ will be measured at baseline and at 4-months follow-up. The YSR will be measured at 4-months follow-up.

#### Secondary outcome measure

The secondary outcome is health-related quality of life measured with the general health perceptions scale of the Child Health Questionnaire Child Form (CHQ-CF87) [31] at baseline and at 4-months follow-up.

#### Socio-demographic characteristics

Socio-demographic questions include gender, age, educational level, country of birth, parents’ country of birth, family structure, employment situation of the parents, and family affluence. Family affluence is measured with the Family Affluence Scale (FAS). The HBSC Family Affluence Scale has been developed by the Health Behaviour in School-Aged Children: WHO Collaborative Cross-National Study (HBSC) to measure the socioeconomic status of adolescents in cross-national surveys [32,33]. The Health Behaviour in School-Aged Children: WHO Collaborative Cross-National Study (HBSC) is an international study carried out by the HBSC International Research Network in collaboration with the WHO Regional Office for Europe. Further information on the study is available from http://www.hbsc.org.

#### Process-evaluation

After the baseline and follow-up questionnaires, the adolescents in the two intervention groups are invited to complete an additional questionnaire. This latter questionnaire provides data on the evaluation of the tailored messages. Topics covered are: appreciation, personal relevance, and perceived usefulness of the information. Questions are asked about: reading the information, reading the reminders, discussing the advice with parents and peers, intention to change their behaviour, the length of the module, using the advice in practice, finding the information interesting, learning new things, and usability. In addition, adolescents are asked to rate the program from 1 (most-negative evaluation) to 10 (most-positive evaluation). Adolescents’ satisfaction with consultation is assessed by 11-items, with answers given on a five-point Likert scale. An extra item is measured to rate the consultation from 1 (most-negative evaluation) to 10 (most-positive evaluation). The nurses complete an evaluation that provides data on: the helpfulness of the information received from the assessment of the referred adolescent, estimation of the problems of the adolescent, action that is taken, and to which professional the adolescent is referred if considered necessary.

#### Power of the study

Sample size was calculated taking into account the design that includes cluster randomisation. We assume an intra-cluster correlation coefficient (ρ) of 0.1. The number of clusters is 60, the power of the study 0.80 and alpha 0.05. With a participation of 85%, and a loss-to-follow-up of 30%, at least 1500 adolescents need to be invited to participate in the study to have a final sample of about 900 adolescents (300 in each group). Assuming a SD of the SDQ total score to be 5.0 [27-29], a difference in mean of 1.6 between the adolescents in the intervention groups and the adolescents in the control group can be established under the assumptions mentioned above. Assuming a SD of the YSR total score (range 0 – 210) to be 20.6 [30], a difference in mean of 6.6 can be established.

#### Statistical analyses

The study assesses the effect of internet-based, tailored messages, and assesses the effect of these messages combined with personal counselling for adolescents at risk of mental health problems. An intention-to-treat analysis will be applied [34].

Multilevel regression analysis will be used to test group differences on the outcome measures [35]. This technique adjusts for the dependency between observations of students from the same class. Linear multilevel analysis is applied for continuous outcome variables and logistic multilevel analysis for dichotomous outcome variables.

Descriptive statistics are used to carry out the process evaluation.

## Discussion

This paper describes the design of a randomised controlled trial on the promotion of health behaviours and the prevention of mental health problems in adolescents.

The study evaluates the effect of internet-based, tailored messages (‘E-health4Uth’), and evaluates the effect of these messages combined with personal counselling for adolescents at risk of mental health problems (‘E-health4Uth + counselling’).

It is hypothesized that at 4-months follow-up adolescents in the ‘E-health4Uth’ group and adolescents in the ‘E-health4Uth + counselling’ group will show fewer mental health problems and less risky behaviour (alcohol and drug use, smoking, safe sex) compared to the control group (‘care as usual’).

Strengths of the study are the randomised controlled design, and providing the intervention within the daily practice of the Dutch Youth Health Care system. This will facilitate implementation of the interventions if found to be effective. Data will be collected in both rural and urban areas of the Netherlands, resulting in a higher level of generalisability. However, because the outcome measures are based on self report, misclassification might occur. In addition, because of including multiple-risk behaviours are included it is not possible to assess each type of behaviour in depth.

In conclusion, this study evaluates the effect of two interventions on the promotion of health behaviour and prevention of mental health problems in adolescents. The results of this study will provide insight into the effectiveness of computer-tailored messages on health behaviours and mental health status, and the effectiveness of these messages combined with personal counselling for adolescents at risk of mental health problems.

## Competing interests

The authors declare that they have no competing interests.

## Authors’ contributions

HR and EA had the original idea for the study and its design, and were responsible for acquiring the study grant. RB further is responsible for the data collection, data analysis and reporting of the study results. RB, EJ and PL provide expert input during the study. HR supervises the study. All authors regularly participated in discussing the design and protocol used in the study. All authors read and approved the final manuscript.

## Pre-publication history

The pre-publication history for this paper can be accessed here:

http://www.biomedcentral.com/1471-2458/12/1083/prepub

## References

[B1] van Dorsselaer S de Looze M Vermeulen-Smit E de Roos S Verdurmen J ter Bogt T Vollebergh W Gezondheid, welzijn en opvoeding van jongeren in Nederland 2009 Utrecht: Trimbos-instituut, Universiteit Utrecht, Sociaal en cultureel planbureau

[B2] Vollebergh WA van Dorsselaer S Monshouwer K Verdurmen J van der Ende J ter Bogt T Mental health problems in early adolescents in the Netherlands: differences between school and household surveys Soc Psychiatry Psychiatr Epidemiol 2006 41 2 156 163 10.1007/s00127-005-0979-x 16453081

[B3] Fergusson DM Woodward LJ Mental health, educational, and social role outcomes of adolescents with depression Arch Gen Psychiatry 2002 59 3 225 231 10.1001/archpsyc.59.3.225 11879160

[B4] Pine DS Cohen P Gurley D Brook J Ma Y The risk for early-adulthood anxiety and depressive disorders in adolescents with anxiety and depressive disorders Arch Gen Psychiatry 1998 55 1 56 64 10.1001/archpsyc.55.1.56 9435761

[B5] Kim-Cohen J Caspi A Moffitt TE Harrington H Milne BJ Poulton R Prior juvenile diagnoses in adults with mental disorder: developmental follow-back of a prospective-longitudinal cohort Arch Gen Psychiatry 2003 60 7 709 717 10.1001/archpsyc.60.7.709 12860775

[B6] Hofstra MB van der Ende J Verhulst FC Child and adolescent problems predict DSM-IV disorders in adulthood: a 14-year follow-up of a Dutch epidemiological sample J Am Acad Child Adolesc Psychiatry 2002 41 2 182 189 10.1097/00004583-200202000-00012 11837408

[B7] Schrijvers C Schoemaker C Spelen met gezondheid. Leefstijl en psychische gezondheid van de Nederlandse jeugd. Volume RIVM Rapport nr. 270232001/2008 2008 Bilthoven: RIVM

[B8] de Wit DJ Adlaf EM Offord DR Ogborne AC Age at first alcohol use: a risk factor for the development of alcohol disorders Am J Psychiatry 2000 157 745 750 10.1176/appi.ajp.157.5.745 10784467

[B9] Koivusilta L Rimpela A Rimpela M Health related lifestyle in adolescence predicts adult educational level: a longitudinal study from Finland J Epidemiol Community Health 1998 52 12 794 801 10.1136/jech.52.12.794 10396520 PMC1756653

[B10] Monshouwer K van Dorsselaer S Verdurmen J ter Bogt T de Graaf R Vollebergh W Cannabis use and mental health in secondary school children. Findings from a Dutch survey Br J Psychiatry 2006 188 148 153 10.1192/bjp.188.2.148 16449702

[B11] Verdurmen J Monshouwer K van Dorsselaer S ter Bogt TWV Alcohol use and mental health in adolescents: interactions with age and gender. Findings from the Dutch 2001 Health Behaviour in School-aged Children Survey J Stud Alcohol 2005 66 605 609 16329458 10.15288/jsa.2005.66.605

[B12] Verdurmen J Monshouwer K van Dorsselaer S Vollebergh W Cannabisgebruik onder adolescenten: gebruikspatronen, achtergrondfactoren en psychosociale problemen 2005 Utrecht: Trimbos-Instituut

[B13] Ministerie van VWS Basistakenpakket jeugdgezondheidszorg 0–19 jaar 2002 Den Haag: Ministerie van Volksgezondheid, Welzijn en Sport

[B14] Dunnink G Advies Extra contactmoment in de leeftijdsperiode 12–19 jaar. Volume Rapport 295001007/2009 2009 Bilthoven: RIVM

[B15] Kreuter MW Farell D Olevitch L Brennan L Tailoring health messages. Customizing communication with computer technology 2000 London: Lawrence Erlbaum Associates

[B16] Kroeze W Werkman A Brug J A systematic review of randomized trials on the effectiveness of computer-tailored education on physical activity and dietary behaviors Ann Behav Med 2006 31 3 205 223 10.1207/s15324796abm3103_2 16700634

[B17] Brug J Oenema A Campbell M Past, present, and future of computer-tailored nutrition education Am J Clin Nutr 2003 77 4 Suppl 1028S 1034S 12663313 10.1093/ajcn/77.4.1028S

[B18] de Vries H Brug J Computer-tailored interventions motivating people to adopt health promoting behaviours: introduction to a new approach Patient Educ Couns 1999 36 2 99 105 10223015 10.1016/s0738-3991(98)00127-x

[B19] Durlak JA Wells AM Evaluation of indicated preventive intervention (secondary prevention) mental health programs for children and adolescents Am J Community Psychol 1998 26 5 775 802 10.1023/A:1022162015815 9861693

[B20] de Nooijer J de Vries NK Monitoring health risk behavior of Dutch adolescents and the development of health promoting policies and activities: the E-MOVO project Health Promot Int 2007 22 1 5 10 10.1093/heapro/dal036 17015406

[B21] de Nooijer J Veling ML Ton A de Vries H de Vries NK Electronic monitoring and health promotion: an evaluation of the E-MOVO Web site by adolescents Health Educ Res 2008 23 3 382 391 10.1093/her/cym086 18187490

[B22] Campbell MK Elbourne DR Altman DG CONSORT statement: extension to cluster randomised trials BMJ (Clinical research ed) 2004 320 7244 1240 1243 10.1136/bmj.328.7441.702 PMC381234 15031246

[B23] Lokale en nationale monitor gezondheidhttp://www.monitorgezondheid.nl

[B24] Joosten-van Zwanenburg E Veilig opgroeien en gezondheid van jongeren in Dordrecht http://www.ggdzhz.nl/pool/1/documents/Veilig%20opgroeien%20en%20gezondheid%20van%20jongeren%20in%20Dordrecht%202008.pdf

[B25] Goodman R Ford T Simmons H Gatward R Meltzer H Using the Strengths and Difficulties Questionnaire (SDQ) to screen for child psychiatric disorders in a community sample Br J Psychiatry 2000 177 534 539 10.1192/bjp.177.6.534 11102329

[B26] Goodman R Meltzer H Bailey V The Strengths and Difficulties Questionnaire: a pilot study on the validity of the self-report version Eur Child Adolesc Psychiatry 1998 7 3 125 130 10.1007/s007870050057 9826298

[B27] Muris P Meesters C van den Berg F The Strengths and Difficulties Questionnaire (SDQ)–further evidence for its reliability and validity in a community sample of Dutch children and adolescents Eur Child Adolesc Psychiatry 2003 12 1 1 8 12601558 10.1007/s00787-003-0298-2

[B28] van Widenfelt BM Goedhart AW Treffers PD Goodman R Dutch version of the Strengths and Difficulties Questionnaire (SDQ) Eur Child Adolesc Psychiatry 2003 12 6 281 289 10.1007/s00787-003-0341-3 14689260

[B29] Janssens A Deboutte D Screening for psychopathology in child welfare: the Strengths and Difficulties Questionnaire (SDQ) compared with the Achenbach System of Empirically Based Assessment (ASEBA) Eur Child Adolesc Psychiatry 2009 18 11 691 700 10.1007/s00787-009-0030-y 19462154

[B30] Achenbach TM Rescorla LA Manual for the ASEBA School-Age Forms & Profiles 2001 Burlington: VT: University of Vermont, Research Center for Childeren, Youth, & Families

[B31] Raat H Landgraf JM Bonsel GJ Gemke RJ Essink-Bot ML Reliability and validity of the child health questionnaire-child form (CHQ-CF87) in a Dutch adolescent population Qual Life Res 2002 11 6 575 581 10.1023/A:1016393311799 12206578

[B32] Currie CE Elton RA Todd J Platt S Indicators of socioeconomic status for adolescents: the WHO Health Behaviour in School-aged Children Survey Health Educ Res 1997 12 3 385 397 10.1093/her/12.3.385 10174221

[B33] Currie C Molcho M Boyce W Holstein B Torsheim T Richter M Researching health inequalities in adolescents: the development of the Health Behaviour in School-Aged Children (HBSC) family affluence scale Soc Sci Med 2008 66 6 1429 1436 10.1016/j.socscimed.2007.11.024 18179852

[B34] Hollis S Campbell F What is meant by intention to treat analysis? Survey of published randomised controlled trials BMJ (Clinical research ed) 1999 319 7211 670 674 10.1136/bmj.319.7211.670 PMC28218 10480822

[B35] Twisk JW Applied multilevel analysis: A Practical Guide for Medical Researchers (Practical Guides to Biostatistics and Epidemiology) 2006 Cambridge: Cambridge University Press

